# Disruption of *pdgfra* alters endocardial and myocardial fusion during zebrafish cardiac assembly

**DOI:** 10.1242/bio.021212

**Published:** 2017-02-06

**Authors:** Suzan El-Rass, Shahram Eisa-Beygi, Edbert Khong, Koroboshka Brand-Arzamendi, Antonio Mauro, Haibo Zhang, Karl J. Clark, Stephen C. Ekker, Xiao-Yan Wen

**Affiliations:** 1 Zebrafish Centre for Advanced Drug Discovery & Keenan Research Centre for Biomedical Science, Li Ka Shing Knowledge Institute, St. Michael's Hospital, Toronto, Ontario, Canada M5B 1T8; 2 Institute of Medical Science, University of Toronto, Toronto, Ontario, Canada M5S 1A8; 3 Collaborative Program in Cardiovascular Sciences, Faculty of Medicine, University of Toronto, Toronto, Ontario, Canada M5S 3E2; 4 Department of Stem Cells and Developmental Biology, Cell Science Research Center. Royan Institute for Stem Cell Biology and Technology, ACECR, Tehran 16635-148, Iran; 5 Department of Medicine & Physiology, Faculty of Medicine, University of Toronto, Toronto, Ontario, Canada M5S 1A8; 6 Department of Biochemistry and Molecular Biology, Mayo Clinic, Rochester, MN 55902, USA

**Keywords:** Pdgfra, Gene trapping, Heart development, Cardiac fusion, Zebrafish

## Abstract

Cardiac development in vertebrates is a finely tuned process regulated by a set of conserved signaling pathways. Perturbations of these processes are often associated with congenital cardiac malformations. Platelet-derived growth factor receptor α (PDGFRα) is a highly conserved tyrosine kinase receptor, which is essential for development and organogenesis. Disruption of *Pdgfrα* function in murine models is embryonic lethal due to severe cardiovascular defects, suggesting a role in cardiac development, thus necessitating the use of alternative models to explore its precise function. In this study, we generated a zebrafish *pdgfra* mutant line by gene trapping, in which the Pdgfra protein is truncated and fused with mRFP (Pdgfra-mRFP). Our results demonstrate that *pdgfra* mutants have defects in cardiac morphology as a result of abnormal fusion of myocardial precursors. Expression analysis of the developing heart at later stages suggested that Pdgfra-mRFP is expressed in the endocardium. Further examination of the endocardium in *pdgfra* mutants revealed defective endocardial migration to the midline, where cardiac fusion eventually occurs. Together, our data suggests that *pdgfra* is required for proper medial migration of both endocardial and myocardial precursors, an essential step required for cardiac assembly and development.

## Abstract (precis)

**Summary:** The molecular mechanisms regulating cardiac fusion are not well understood. Here, we show that platelet-derived growth factor receptor alpha is essential for normal endocardial and myocardial fusion during zebrafish development.

## INTRODUCTION

Cardiac development in vertebrates is a precisely coordinated process involving multiple regulatory pathways that control specification and differentiation of cardiac precursors, followed by morphogenesis of the multi-chambered heart and maintenance of cardiac function. Perturbations of these processes, by virtue of mutations and/or environmental toxins/teratogens, can give rise to abnormal cardiac morphogenesis, and are often associated with congenital/fetal cardiac malformations in patients. Although a number of conserved signaling pathways have been implicated in cardiac morphogenesis in various vertebrate models, the precise function of many genes remains elusive (Srivastava and Olson, 2000; Bruneau, 2008; Epstein, 2010; Mahler and Butcher, 2011).

In vertebrates, heart tube assembly begins with movement of the bilateral cardiac fields to either side of the embryonic midline. As cardiogenesis proceeds, myocardial precursors merge together and surround the centrally located endocardial precursors, through a process called cardiac fusion. Cardiac fusion forms an intermediate structure, which gradually transforms into a primitive and thin-layered heart tube consisting of an inner endocardium and an outer myocardium. Subsequently, the heart tube forms different chambers; the main ones being the atrium and ventricle (Glickman and Yelon, 2002; Harvey, 2002; Moorman and Christoffels, 2003). Unlike traditional vertebrate models, zebrafish can survive during embryogenesis in the absence of cardiac output and circulation due to passive diffusion of oxygen across their skin (Stainier, 2001). This feature facilitates functional characterization of cardiovascular genetic mutations that are otherwise embryonically lethal. Furthermore, the rapid, *ex utero* embryonic development and relatively transparent nature make zebrafish an excellent model for cardiovascular research (Kimmel et al., 1995; Chen et al., 1996; Briggs, 2002; Beis and Stainier, 2006).

The platelet derived growth factor receptor alpha (*PDGFRα*) gene encodes a highly conserved tyrosine kinase receptor. In vertebrates, binding of PDGF ligands to PDGF receptors stimulates their tyrosine kinase activity and results in transphosphorylation of specific tyrosine residues, which can act as binding sites for intracellular signaling molecules (Hoch and Soriano, 2003). In general, PDGFRα-mediated signaling is essential for embryonic development and organogenesis as it has been implicated in mediating differentiation, migration and function of specialized mesenchymal cells (Stephenson et al., 1991; Boström et al., 1996; Soriano, 1997; Fruttiger et al., 1999; Gnessi et al., 2000; Karlsson et al., 2000; Tallquist and Soriano, 2003).

Absence of PDGFRα in mouse *Patch* mutants results in cardiac defects, including enlarged hearts (Orr-Urtreger et al., 1992), septal defects and reduced myocardial wall thickness (Morrison-Graham et al., 1992; Schatteman et al., 1995), dilated hearts and abnormal valves (Schatteman et al., 1992). Likewise, there is preliminary evidence of cardiac defects in a zebrafish *pdgfra* mutant, as well as Mirn140-mediated attenuation of Pdgfra signaling in zebrafish (Eberhart et al., 2008). In humans, allelic variations in *PDGFRα* have been associated with total anomalous pulmonary venous return (TAPVR), a congenital malformation of the heart that results in abnormal pulmonary venous drainage into the right atrium instead of the left atrium (Bleyl et al., 2010). Despite this strong association of *PDGFRα* genetic variation and human disease, the precise role of PDGFRα in cardiac development remains unclear.

In this study, we employed the well-defined GBT-RP2.1 (RP2) protein trap vector for mutagenesis, a system that efficiently disrupts endogenous genes by truncating the native transcript and simultaneously tagging the truncated product with monomeric red fluorescent protein (mRFP), thus allowing for spatio-temporal labeling of the chimeric protein (Clark et al., 2011). We report a mutant line, designated as GBT1300, which expresses mRFP in the developing zebrafish cardiovascular system. In GBT1300, the RP2 transgene was inserted in intron 16 of the *pdgfra* gene. We demonstrate that loss of *pdgfra* function gives rise to a defective cardiac morphology as a result of abnormal fusion of myocardial precursors. Expression analysis of isolated hearts at 30 h post fertilization (hpf) suggested that Pdgfra-mRFP is expressed in the endocardium. Further examination of *pdgfra* mutants at early stages revealed defective endocardial migration to the midline, where cardiac assembly eventually occurs. Together, our data suggests that loss of Pdgfra function results in defective medial migration of both myocardial and endocardial precursors, resulting in abnormal cardiac morphology.

## RESULTS

### RP2 transposon insertion mediates loss of *pdgfra* function in zebrafish

**Fig. 1.**
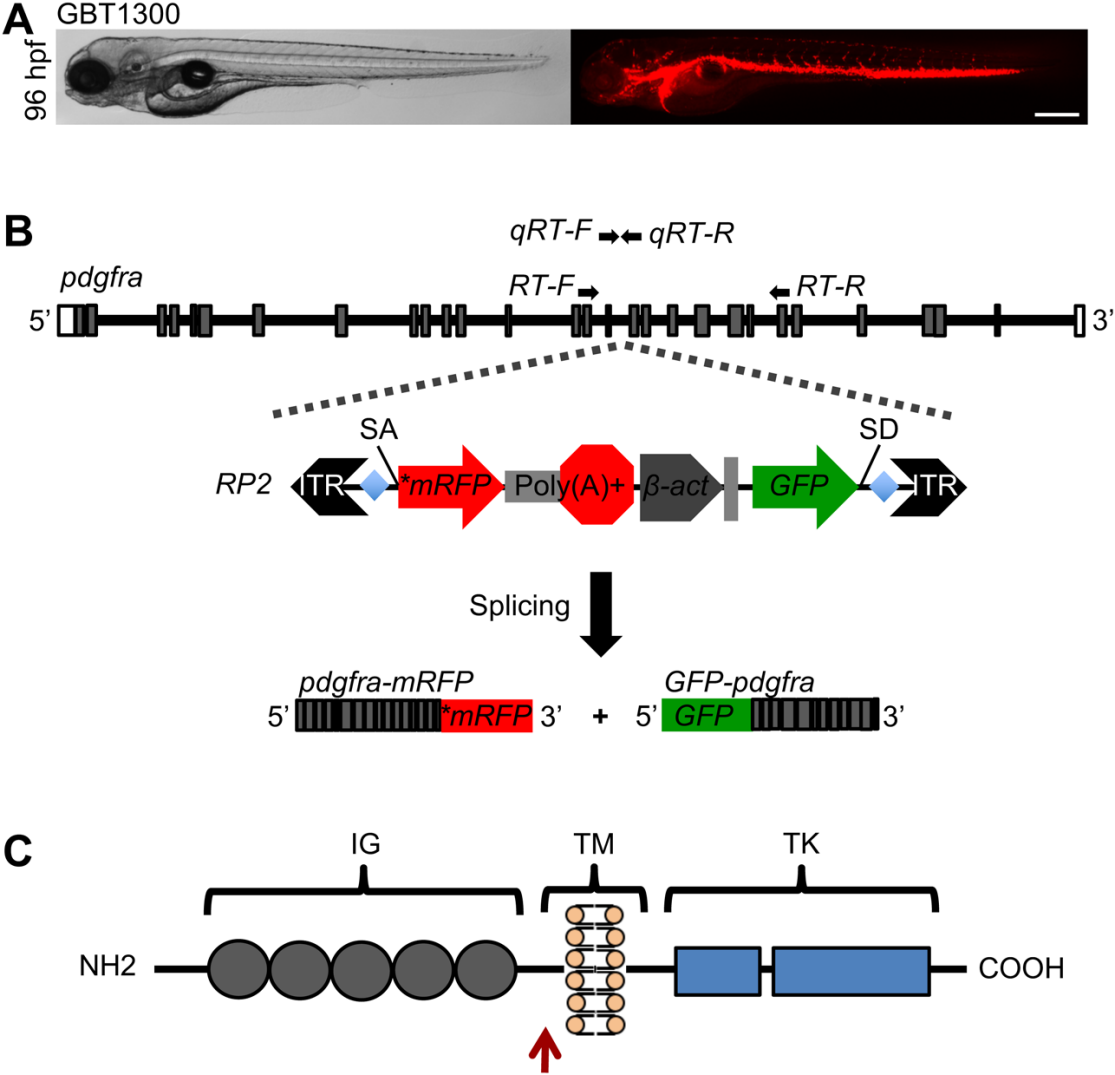
**RP2 Transposon integration disrupts *pdgfra* gene.** (A) Bright field (left) and fluorescent (right) images of heterozygous GBT1300 larva showing mRFP expression pattern at 96 hpf. (B) Schematic representation of zebrafish *pdgfra* gene showing the locus of RP2 insertion in intron 16, and the resulting truncated *pdgfra-mRFP* and *GFP-pdgfra* fusion transcripts. Gray boxes indicate exons; lines indicate introns. Primers used for reverse transcription (RT)- and quantitative real-time (qRT)-PCR are denoted. (C) Schematic drawing of zebrafish Pdgfra protein domains, showing the site of mRFP fusion (red arrow). IG, immunoglobulin; TM, transmembrane; TK, tyrosine kinase. Scale bar: 500 μm in A.

The ability to identify, in real time, the expression of a gene is helpful for elucidating its tissue-specific function. Microinjection of newly fertilized zebrafish embryos with the RP2 vector yielded 350 chimeric F_0_ fish, of which 51 produced stable gene-insertional mutants with robust mRFP expression patterns detected in various tissues and cell-types. In this study, we sought to expound on an insertional line we designated as GBT1300, in which a distinct mRFP expression pattern along the cardiovascular system was evident (Fig. 1A).

Next, we proceeded to identify the genomic locus of transposon integration in the GBT1300 line. We employed both inverse PCR and TAIL PCR strategies on genomic DNA harvested from GBT1300 fish, and revealed an RP2 insertion site in intron 16 of *pdgfra*. As a result of the RP2 insertion, two transcripts were made: a truncated *pdgfra-mRFP* fusion transcript produced by the endogenous *pdgfra* promoter, and a ubiquitously expressed *GFP-pdgfra* fusion transcript produced by the RP2 ß-actin promoter (Fig. 1B). The *pdgfra* gene encodes a highly conserved tyrosine kinase receptor, Pdgfra, consisting of extracellular immunoglobulin domains, a transmembrane domain, and cytoplasmic tyrosine kinase domains (Fig. 1C) (Yarden et al., 1986; Claesson-Welsh et al., 1989; Matsui et al., 1989). Sequence analyses revealed that the predicted GBT1300 transcript lacks the tyrosine kinase domains required for downstream signaling processes.

**Fig. 2.**
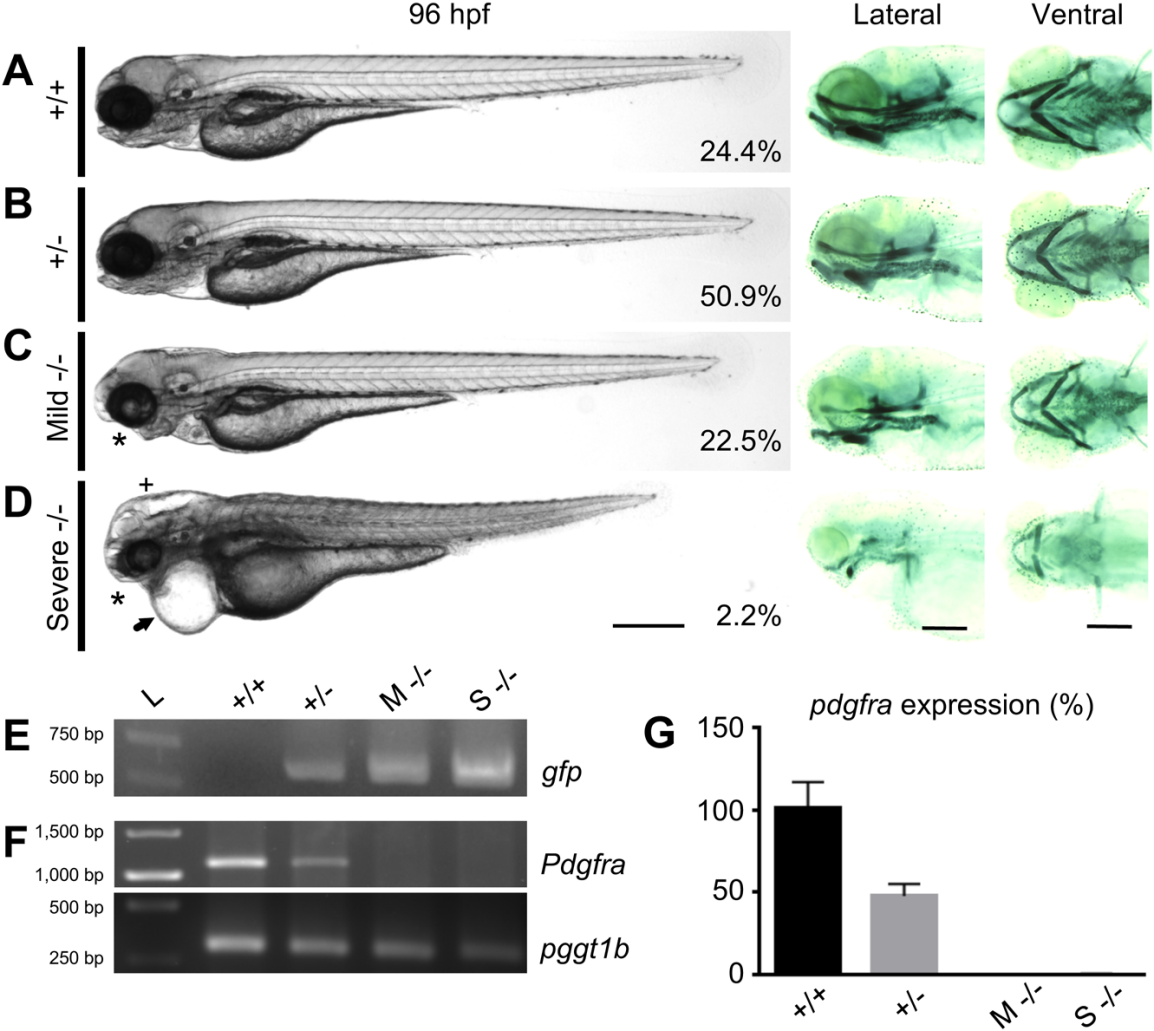
**General phenotype analyses in *pdgfra* loss-of-function mutants.** Panels showing bright field (left), and Alcian Blue-stained cartilaginous tissue (right) of WT (+/+) (A), heterozygous (+/−) (B), mild homozygous (Mild/M −/−) (C), and severe homozygous (Severe/S −/−) (D) GBT1300 siblings at 96 hpf. The percentage of fish in each group is indicated (*n*=275). While +/+ and +/− larvae show no visible abnormalities, mild −/− mutants exhibit craniofacial defects (asterisk) and abnormal swim bladder inflation. Severe −/− mutants demonstrate severe developmental defects, including craniofacial defects (asterisk), pericardial edema (arrow), hydrocephalus (cross), smaller body axis elongation, and abnormal cardiac and skeletal muscle. (E) PCR indicating the presence of *gfp* in +/−, mild −/− and severe −/−, but not in +/+ fish. (F) RT-PCR demonstrating reduction in endogenous *pdgfra* transcripts in mild −/− and severe −/−, compared to +/+ and +/− fish at 96 hpf. *pggt1b* used as a control for RT-PCR. (G) Relative amounts of *pdgfra* transcript confirming the loss of WT *pdgfra* transcripts in both mild −/− and severe −/− mutants at 96 hpf through qRT-RCR (*P*≤0.01; *n*=25/group; unpaired *t* test, error bars indicate s.e.m.). Scale bar: 250 μm. Anterior is to the left.

We subsequently studied the association of the mutant phenotype with the RP2 transgene insertion. An in-cross of GBT1300 yielded 24.4% wild-type (WT), 50.9% heterozygous, 22.5% mild homozygous, and 2.2% severe homozygous fish (*n*=275) (Fig. 2A-D). Given that both mice and zebrafish with loss-of-function mutations in PDGFRα show defects in palatogenesis (Soriano, 1997; Tallquist and Soriano, 2003; Eberhart et al., 2008), we examined the GBT1300 mutants for craniofacial abnormalities. Similarly, craniofacial defects in both mild and severe GBT1300 homozygous larvae were detected and further confirmed using Alcian Blue staining (Fig. 2A-D). The craniofacial abnormalities in homozygous mutants, collected from a cross of *Tg(GBT1300;sox10:EGFP)* with GBT1300, were preceded by failure of neural crest (Sox10-positive) cells to reach the oral ectoderm at 24 hpf (Fig. S1) consistent with previous findings (Eberhart et al., 2008). Along with the craniofacial deformities, severe homozygous mutants developed other defects, including abnormal cardiac and skeletal, pericardial edema, short body axis and hydrocephalus (Fig. 2D).

Injection of WT *pdgfra* mRNA into newly fertilized eggs, collected from a heterozygous GBT1300 cross, resulted in a reduction of mutant phenotype [injected 10.9% (*n*=128) and uninjected 24.3% (*n*=189)]. This data provided conformation that the RP2 insertion in *pdgfra* is responsible for the mutant phenotype. It is important to note that injection of *pdgfra* mRNA into WT and heterozygous embryos often resulted in over-extended jaws and cyclopia (data not shown), suggesting a dose dependent requirement for *pdgfra*, as previously proposed (Eberhart et al., 2008). The phenotypic similarity of our GBT1300 line with previous reports corroborates that the employed RP2-mediated mutagenesis approach efficiently disrupted Pdgfra function.

To rule out additional RP2 insertion sites that may have non-specifically contributed to the severe phenotype, we performed PCR analyses on genomic DNA extracted from WT, heterozygous and homozygous siblings to amplify the GFP sequence of the RP2 transposon. Unlike in homozygous and heterozygous fish, WT siblings showed no amplification of the GFP sequence (Fig. 2E), suggesting that no additional RP2 insertions were present. Next, we performed RT-PCR analyses to confirm that endogenous *pdgfra* transcripts at the site of RP2 insertion are absent. The results demonstrated that the *pdgfra* transcripts in both mild and severe homozygotes were absent compared to their WT and heterozygous siblings (Fig. 2F). The reduction in the mRNA levels of *pdgfra* was further validated using the qRT-PCR method (*P*≤0.01; Fig. 2G). Moreover, sequence analysis of the fusion *pdgfra-mRFP* cDNA revealed that the mRFP fusion was in frame (data not shown), suggesting that the observed mRFP pattern is directly associated with Pdgfra expression. Taken together, these results attest to the efficiency of the RP2 insertion method in disrupting *pdgfra* gene function and labeling the truncated protein product with mRFP.

### Loss of *pdgfra* function is associated with abnormal cardiac morphology

**Fig. 3.**
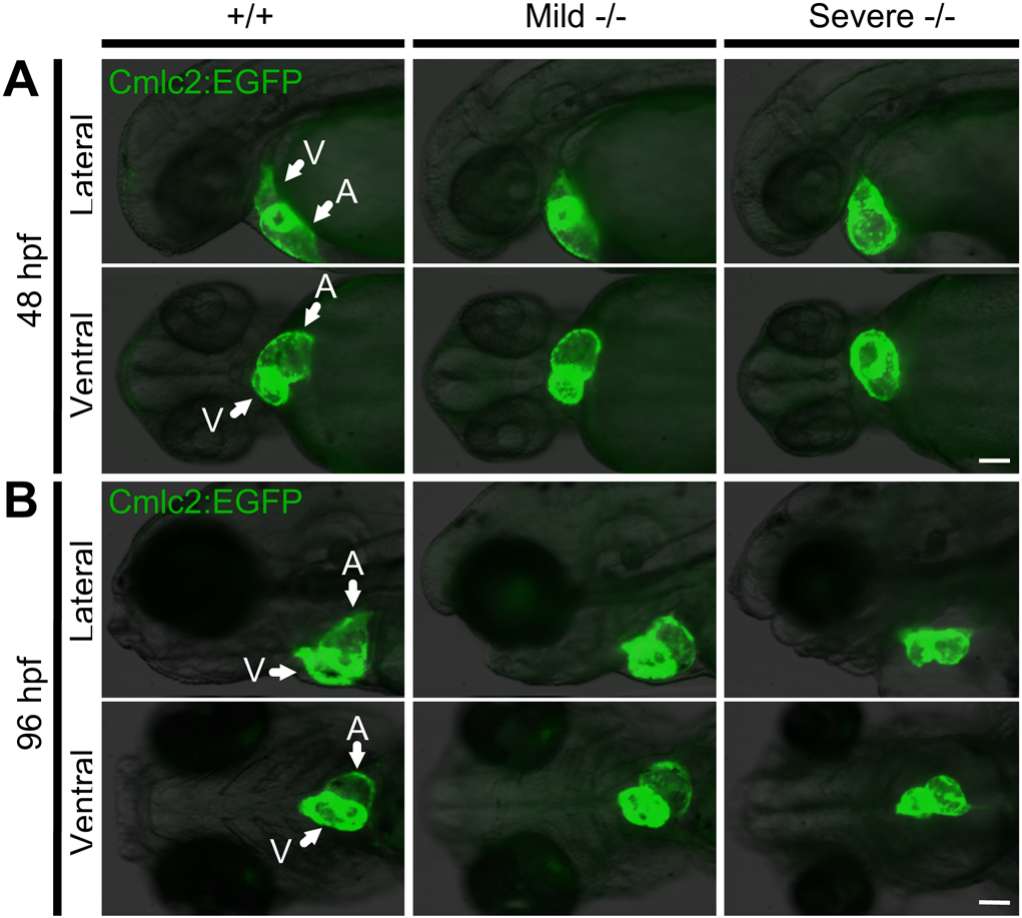
**Pdgfra homozygous mutants exhibit abnormal cardiac phenotypes.** An incross of the *pdgfra* gene-trapped strain carrying a *Tg(cmlc2:EGFP)* transgene was used to investigate the cardiac phenotype. (A) At 48 hpf, mild and severe −/− mutants present with abnormal cardiac looping; severe −/− embryos also develop an enlarged atrium and pericardial edema. (B) At 96 hpf, the hearts of mild −/− mutants remain incompletely looped, while severe −/− mutants present with collapsed chambers and pericardial edema. Arrows pointing at ventricle (V) and atrium (A). The fish cranium is on the left. Scale bar: 100 μm.

To characterize the cardiac defects associated with loss of *pdgfra* function, we crossed heterozygous GBT1300 with *Tg(GBT1300;cmlc2:EGFP)*, in which the *cmlc2* promoter marks cardiomyocytes (Huang et al., 2003). Although the RP2 system induces ubiquitous GFP expression as a marker for transposon insertion (Clark et al., 2011), the GFP expression in our GBT1300 line was very weak likely due to non-sense mediated mRNA decay of the 3′ exon trapped chimeric RNA. This enabled us to cross the GBT1300 line with other transgenic lines expressing GFP strongly in specific tissues. When compared to WT siblings, both mild and severe homozygotes presented with abnormal cardiac looping phenotypes; however, the severe homozygotes also manifested enlarged atrium and pericardial edema at 48 hpf (*n*≥5/group) (Fig. 3A; Movies 1, 2 and 3). In WT larvae, the atrium was positioned at the same anterior-posterior level as the ventricle at 96 hpf. By contrast, the hearts in the mild homozygotes remained incompletely looped, and severe homozygotes presented with collapsed chambers and pericardial edema at 96 hpf (Fig. 3B; Fig. S2). Interestingly, about 10% of heterozygous larvae also showed incomplete heart tube looping between 48 and 96 hpf, likely due to a dose-dependent requirement for *pdgfra*; however, this defect was not as prevalent as in the homozygous siblings (data not shown). Together, our data suggests that loss of *pdgfra* function is associated with abnormal cardiac morphology.

Concomitant with cardiac defects, the severe homozygotes also demonstrated absence of blood circulation at all stages of development, which was confirmed through fluorescent microangiography at 72 hpf (*n*≥8/group; Fig. S3). The circulatory defects, however, appeared not to be due to defective erythropoiesis/hematopoiesis, as hemoglobin-specific OD-staining revealed presence of erythrocytes in these fish (*n*≥3/group; Fig. S4). These results suggest that the impaired circulation in severe homozygous mutants was likely due to the abnormal cardiac phenotype, as opposed to inhibition of hematopoiesis. Although mild homozygous mutants demonstrated abnormal cardiac morphologies, blood circulation was present; nevertheless, mild mutants died at around 14 days post fertilization (dpf), likely due to the jaw defects and lack of proper swim bladder inflation which prevented the mutants from feeding and swimming.

### Pdgfra is required for proper fusion of myocardial precursors

**Fig. 4.**
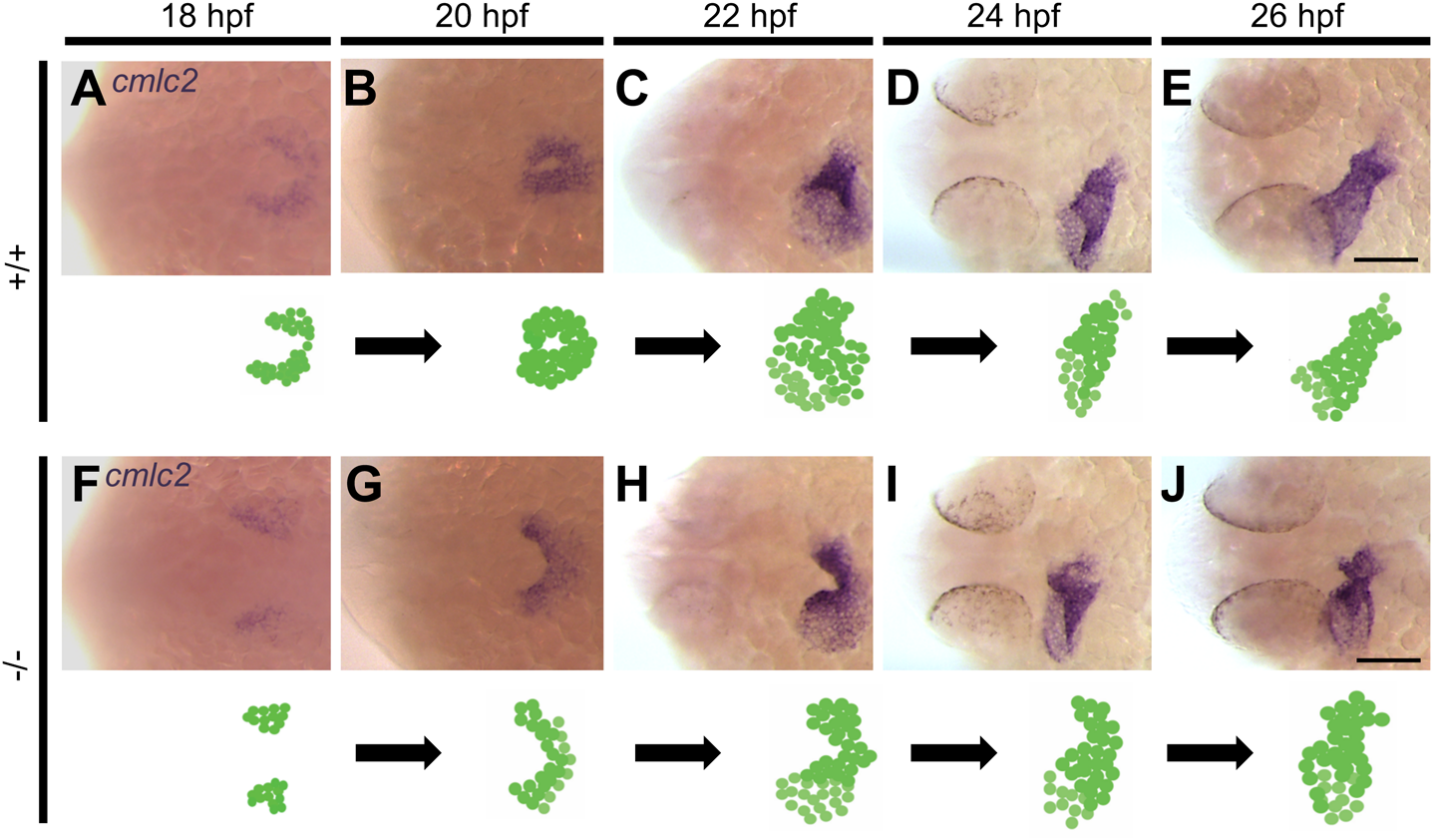
**Homozygous *pdgfra* mutants demonstrate abnormal cardiac fusion.** Representative photomicrographs of whole-mount *in situ* hybridization using the *cardiac myosin light chain 2* (*cmlc2*) riboprobe to label myocardial cells. In +/+ embryos, the posterior regions of the bilateral cardiac fields interact during cardiac fusion at 18 hpf (A), followed by interaction between the anterior regions at 20 hpf (B). At 22 hpf, the cardiac tube forms and symmetry is broken by leftward displacement (jogging) (C). At 24 (D) and 26 (E) hpf, the cardiac tube elongates. In −/− mutants, cardiac fusion is delayed at 18 hpf (F). Interaction between the posterior regions occurs at 20 hpf (G). At 22 hpf, the anterior regions of the bilateral cardiac fields fail to fuse (H). By 24 (I) and 26 (J) hpf, the anterior portions of the bilateral cardiac fields begin to come into contact. Dorsal views are shown, with the fish cranium on the left. An illustration of the developing heart is shown at the bottom of each figure. Scale bar: 100 μm.

Given that zebrafish have a functional cardiac output as early as 24 hpf, we proceeded to identify the exact developmental timing during which the cardiac phenotype arises in the GBT1300 line. Embryos obtained from a cross of heterozygous GBT1300 were stained for *cmlc2* expression through whole-mount ISH, using a riboprobe against *cmlc2* mRNA at different stages (18, 20, 22, 24 and 26 hpf; *n*=24/stage). In WT embryos, cardiac fusion was initiated when the posterior regions of the bilateral cardiac fields came into contact at 18 hpf (Fig. 4A). At 20 hpf, interaction between the anterior regions of the bilateral fields was observed, resulting in the formation of a cardiac cone (Fig. 4B). At 22 hpf, the cardiac cone had telescoped outward to form the cardiac tube and cardiac symmetry was broken by leftward displacement (jogging) of the heart tube (Fig. 4C). By 24 and 26 hpf, the cardiac tube had extended and assumed an elongated cylindrical shape (Fig. 4D,E). These observations of early zebrafish cardiac development are consistent with previous studies (Bakkers et al., 2009; Holtzman et al., 2007; Stainier, 2001).

In contrast to WT embryos, *pdgfra* homozygous mutants demonstrated a delay in cardiac fusion at 18 hpf (Fig. 4F). By 20 hpf, fusion of the posterior regions of the bilateral fields was observed (Fig. 4G). At 22 hpf, the anterior regions of the bilateral cardiac fields failed to fuse, resulting in persistent cardiac fields fused only at the posterior ends (Fig. 4H). Although the anterior regions of the bilateral cardiac fields remained separate at 22 hpf, more myocardial cells were observed in the left region than the right, suggesting that leftward jogging was initiated (Fig. 4H). Between 24 and 26 hpf, anterior fusion of the myocardium is observed (Fig. 4I,J), whereas cardiac morphology remained abnormal. The abnormal cardiac fusion phenotype was observed in all homozygous embryos, suggesting that both mild and severe mutants require *pdgfra* for normal cardiac fusion. Collectively, these expression-based data suggest that embryonic loss of *pdgfra* function gives rise to delayed fusion of the anterior regions of the bilateral cardiac fields, resulting in abnormal cardiac morphology.

### The delay in anterior fusion of the bilateral cardiac fields disrupts the development of cardiac chambers in *pdgfra* mutants

**Fig. 5.**
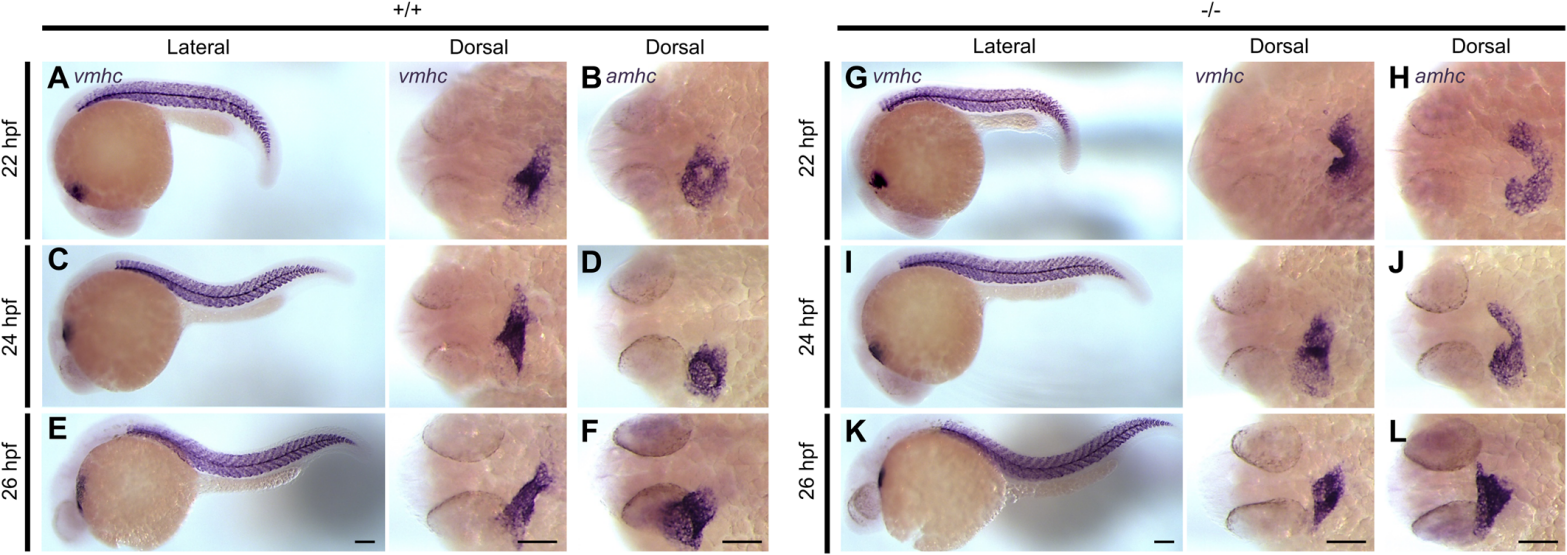
**Delay in anterior cardiac fusion disrupts the development of cardiac chambers in *pdgfra* mutants.** Representative images of embryos subjected to whole-mount *in situ* hybridization with riboprobes against *ventricular myosin heavy chain* (*vmhc*) and *atrial myosin heavy chain* (*amhc*). In +/+ embryos, the ventricular cells are observed at the apex of the cone (A) and the atrial cells at its base (B) at 22 hpf. At 24 and 26 hpf, the ventricles elongates (C and E), and the atria become cohesive and tubular (D and F). In −/− mutants both the ventricular (G) and atrial (H) regions of the cardiac cone remain incompletely fused at 22 hpf. At 24 to 26 hpf, the anterior portions of the bilateral cardiac fields begin to come into contact; however, the ventricles (I and K) and the atria (J and L) appear shorter and wider. Lateral and dorsal views are shown, with the fish cranium on the left. Scale bar: 100 μm.

Using molecular markers, atrial and ventricular cell lineages can be distinguished before the chambers mature to assume morphologically distinct characteristics (Yelon et al., 1999). To investigate whether the delay in anterior fusion of the bilateral cardiac fields affects the development of heart chambers, we evaluated the spatio-temporal expression pattern of the ventricle and atrial specific genes *vmhc* and *amhc*, respectively. Whole-mount ISH was performed on embryos obtained from a cross of heterozygous GBT1300 using riboprobes against *vmhc* and *amhc* mRNA at 22, 24 and 26 hpf (*n*=24/stage for each probe). In WT embryos, the ventricular cells were observed at the apex of the cone (Fig. 5A), and the atrial cells at its base (Fig. 5B) at 22 hpf. At 24 and 26 hpf, the ventricles had elongated normally (Fig. 5C,E), and the atria became cohesive and tubular (Fig. 5D,F).

In contrast to WT embryos, homozygous mutants demonstrated striking differences in chamber morphology. At 22 hpf, both the ventricular and atrial regions of the cardiac cone remained incompletely fused as a result of failed fusion of the anterior regions of the bilateral cardiac fields (Fig. 5G,H). At around 24 and 26 hpf, contact between anterior portions of the chambers was observed. However, the ventricles in homozygous embryos appeared shorter and wider than that of their WT siblings (Fig. 5I,K), and cells in the atria exhibited subtle expansion and widening (Fig. 5J,L). Together, these data suggest that although atrial and ventricular fates are assigned in *pdgfra* mutants, delayed interaction between anterior regions of the bilateral cardiac fields results in abnormal atrial and ventricular development.

### Pdgfra is expressed in the midline during cardiac assembly

**Fig. 6.**
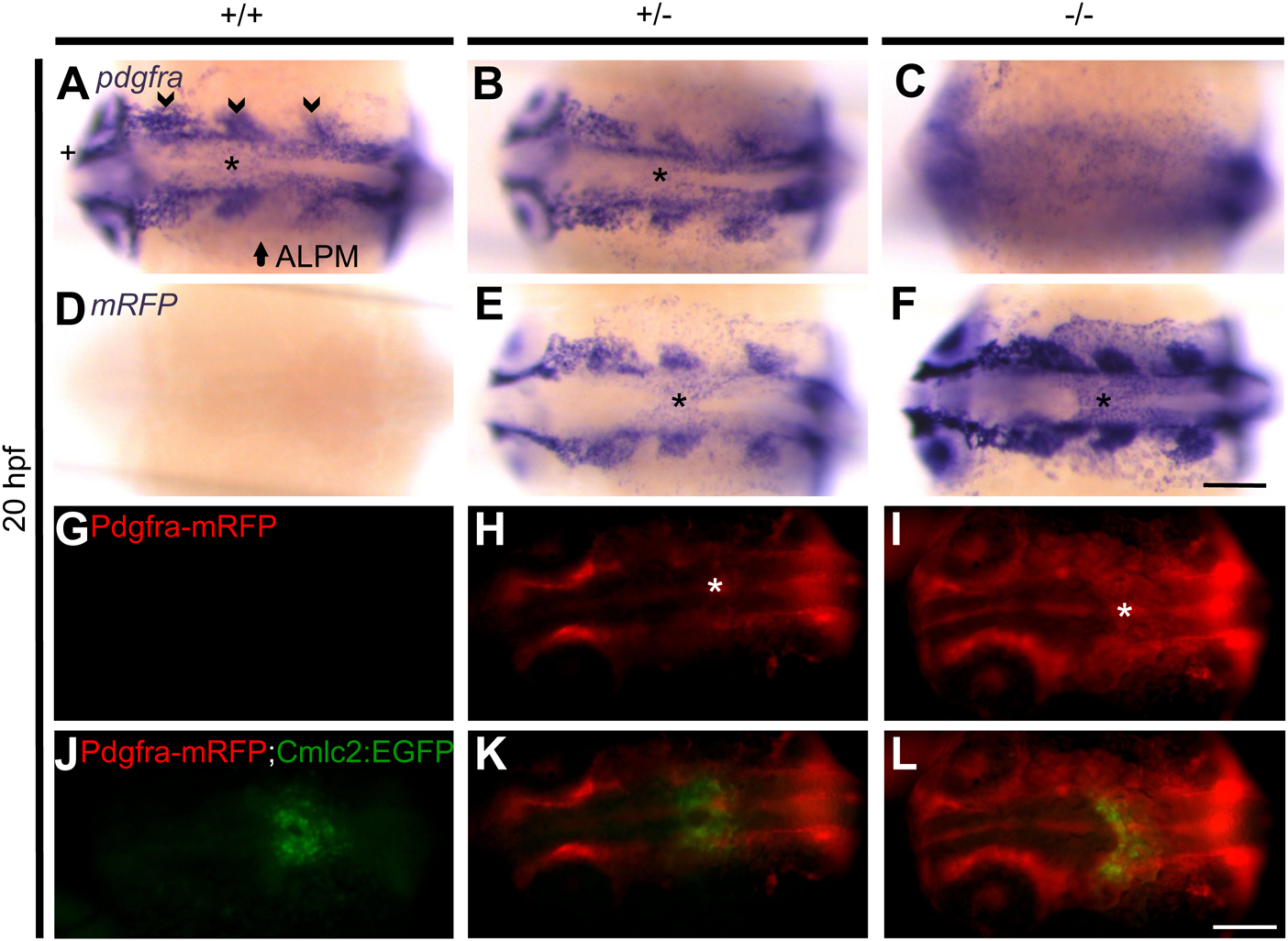
**Pdgfra is expressed in the midline during cardiac assembly.** Representative photomicrographs of whole-mount *in situ* hybridization using *pdgfra* and *mRFP* riboprobes at 20 hpf (A-F). In +/+ (A) and +/− (B) embryos expression of *pdgfra* is observed in different tissues, including the developing cranial ganglia (arrow head), anterior lateral plate mesoderm (ALPM) (arrow), optic cup (cross), and the midline (asterisk, also in panels E and F). No specific *pdgfra* expression is detected in −/− embryos (C). Expression of *mRFP* is not detected in +/+ embryos (D). On the other hand, *mRFP* expression in +/− (E) and −/− (F) embryos is similar to *pdgfra* expression. Representative images of 20 hpf live GBT1300 siblings carrying a *Tg(cmlc2:EGFP)* transgene (G-L). No Pdgfra-mRFP expression is observed in +/+ embryos (G). On the other hand, +/− (H) and −/− (I) siblings show Pdgfra-mRFP expression similar to the mRNA expression data, including midline expression where cardiac assembly occurs (asterisk). Complete cardiac fusion is observed in +/+ (J) and +/− (K) embryos, while homozygous mutants demonstrated cardiac fusion only at the posterior end (L). Dorsal views are shown, with the fish cranium on the left. Scale bar: 75 μm (A-F) and 250 μm (G-L).

To further examine how Pdgfra influences cardiac assembly, we examined the expression pattern of endogenous *pdgfra* mRNA using a *pdgfra* riboprobe. Whole-mount ISH was performed on 20 hpf embryos collected from a heterozygous GBT1300 cross. WT and heterozygous embryos demonstrated *pdgfra* expression in different tissues, including the developing cranial ganglia, anterior lateral plate mesoderm (ALPM) and optic cup, as previously described (Liu et al., 2002). Interestingly, *pdgfra* expression was also detected in the midline where cardiac assembly occurs (Fig. 6A,B). As predicted, homozygous embryos did not illustrate tissue-specific expression of *pdgfra*. Instead, the expression was scattered, likely due to partial hybridization of the *pdgfra* riboprobe to both *pdgfra-mRFP* fusion mRNA and the ubiquitously expressed *GFP-pdgfra* fusion mRNA (Fig. 6C). Whole-mount ISH was also performed using an *mRFP* riboprobe to investigate whether the *pdgfra-mRFP* fusion mRNA expression pattern is similar to that of *pdgfra*. As expected, WT embryos did not express *mRFP* (Fig. 6D); however, heterozygous embryos illustrated *mRFP* expression similar to the expression pattern of *pdgfra* (Fig. 6B,E), while the homozygous expression pattern was stronger (Fig. 6F). This data verified that the *pdgfra-mRFP* fusion mRNA is made when and where the endogenous gene's mRNA is transcribed.

Next, we sought to validate if the Pdgfra-mRFP fusion protein is also locally produced, and whether the expression in the midline co-localizes with the myocardium. To do this, embryos collected from a cross of heterozygous GBT1300 with *Tg(GBT1300;cmlc2:EGFP)* were imaged at 20 hpf, and the expression pattern was analyzed. As expected, WT embryos did not express the Pdgfra-mRFP fusion protein (Fig. 6G); however, heterozygous and homozygous embryos demonstrated mRFP expression similar to the mRNA expression data, including midline expression where cardiac assembly occurs (Fig. 6H,I). The consistent expression patterns between endogenous *pdgfra*, *pdgfra-mRFP* fusion mRNA and Pdgfra-mRFP fusion protein indicated local retention of the fusion protein. Moreover, complete cardiac fusion was observed in WT and heterozygous embryos (Fig. 6J,K), while homozygous mutants demonstrated cardiac fusion only at the posterior end (Fig. 6L), consistent with the *cmlc2* ISH data. Although the Pdgfra-mRFP is expressed in the midline where cardiac fusion occurs, it was not clear from the whole embryo images whether mRFP-positive cells co-localized with Cmlc2-derived EGFP-positive cells (myocardium) due to the dispersed nature of Pdgfra expression and excessive background.

### Analysis of the developing heart suggested expression of Pdgfra-mRFP in the endocardium

**Fig. 7.**
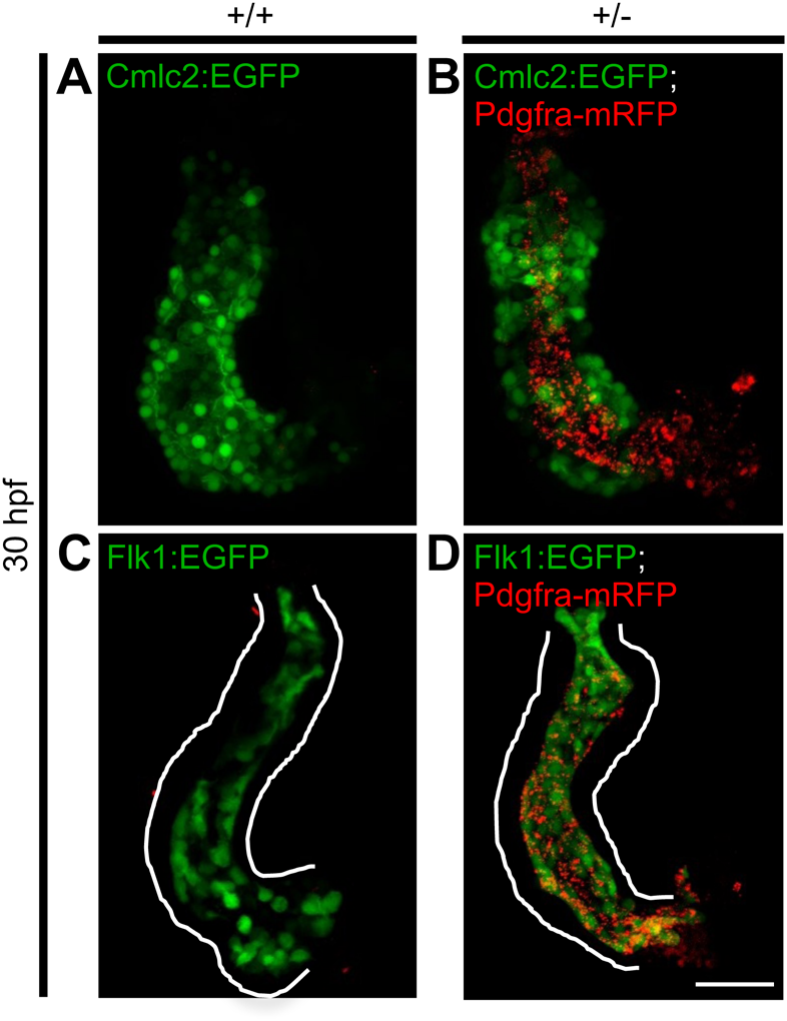
**Analysis of the developing heart suggested expression of Pdgfra-mRFP in the endocardium rather than the myocardium.** Dissected hearts from +/+ and +/− GBT1300 siblings carrying either a *Tg(cmlc2:EGFP)* or *Tg(flk1:EGFP)* transgene were used to investigate the expression pattern of the fusion Pdgfra-mRFP at 30 hpf. Cmlc2 positive +/+ embryos show no Pdgfra-mRFP expression in the heart (A), while +/− embryos exhibit Pdgfra-mRFP expression internal to the myocardium (B). Flk positive +/+ embryos show no Pdgfra-mRFP expression in the heart (C), while +/− embryos suggested co-localization of Pdgfra-mRFP with the endocardium (D). Scale bar: 50 μm.

Since Pdgfra-mRFP-positive tissue(s) surrounding the developing heart may interfere with cardiac expression analyses, we isolated hearts from embryos at 30 hpf, a stage in which the heart is intact and can readily be dissected. Hearts of WT and heterozygous embryos, collected from a cross of heterozygous GBT1300 and *Tg(cmlc2:EGFP)*, were isolated to check whether Pdgfra-mRFP expression co-localizes with Cmlc2-positive cells (myocardium). As expected, Cmlc2-positive WT embryos did not express mRFP in the heart (Fig. 7A). Interestingly, Pdgfra-mRFP was not expressed by Cmlc2-positive cells in heterozygous hearts, instead, mRFP expression was observed internal to the myocardium (Fig. 7B). Since the heart is composed of an outer myocardium and an inner endocardium (Stainier, 2001; Glickman and Yelon, 2002), we next crossed heterozygous GBT1300 with *Tg(flk1:EGFP)*, in which *flk1* promoter marks endothelial cells (Jin et al., 2005). No mRFP expression was observed in the hearts of Flk1-positive WT embryos, as predicted (Fig. 7C); however, heterozygous hearts appeared to co-express Pdgfra-mRFP- and Flk1-derived EGFP (endocardium) (Fig. 7D). These data suggested that Pdgfra-mRFP is expressed in the endocardium at 30 hpf, and thus proposed that the endocardium may also be disrupted during cardiac fusion in *pdgfra* mutants.

### Endocardial migration to the midline is defective in *pdgfra* mutants

**Fig. 8.**
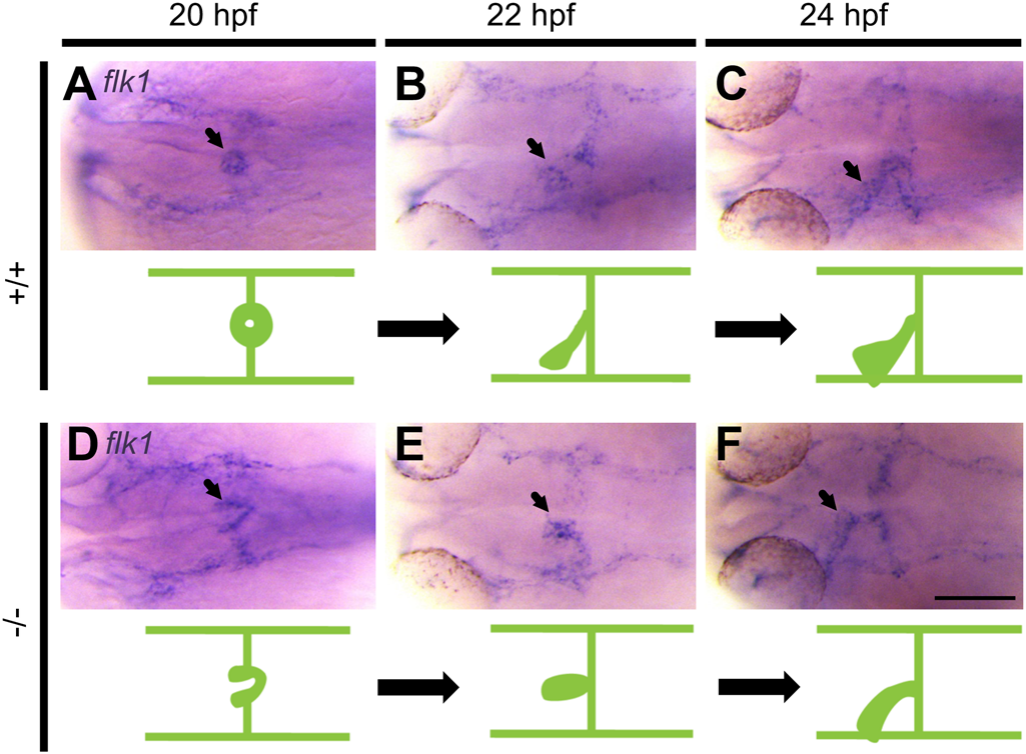
**Migration of endocardial precursors to the midline is abnormal in *pdgfra* mutants.** Representative photomicrographs of whole-mount *in situ* hybridization using *fetal liver kinase 1* (*flk1*) riboprobe. In +/+ embryos, endocardial precursors migrate and fuse at the midline forming a ring-like structure at 20 hpf (A), followed by elongation and leftward movement at 22 (B) and 24 (C) hpf. In −/− mutants, endocardial precursors form a V-like structure at 20 hpf (D). Endocardial cells reach the midline and begin to elongate at 22 hpf (E). Elongation continues with abnormal leftward movement at 24 hpf (F). Arrows point to endocardium. Dorsal views are shown, with the fish cranium on the left. An illustration of the developing heart is shown at the bottom of each figure. Scale bar: 100 μm.

Since Pdgfra-mRFP fusion protein was detected in the endocardium of the developing heart at 30 hpf, we sought to investigate whether the endocardium is defective at early stages in *pdgfra* mutants. In zebrafish, endocardial precursors migrate from either side of the ALPM to the midline shortly before fusion of myocardial precursors (Bussmann et al., 2007; Holtzman et al., 2007). To investigate whether endocardial migration to the midline is defective in *pdgfra* mutants, we performed whole-mount ISH on embryos collected from a heterozygous GBT1300 cross using a *flk1* riboprobe (*n*=24/stage). In WT embryos, endocardial precursors migrated and fused at the midline forming a ring-like structure at 20 hpf (Fig. 8A). At 22 and 24 hpf, elongation and leftward movement of the endocardium was detected (Fig. 8B,C). The observed endocardial morphogenesis in WT embryos are consistent with previous findings (Bussmann et al., 2007). In contrast to WT embryos, *pdgfra* mutants showed abnormal endocardial migration to the midline at 20 hpf. Instead of forming a ring, endocardial precursors formed a V-shaped structure at the midline (Fig. 8D). At 22 hpf, endocardial cells reached the midline and elongation was initiated, however, leftward movement was not observed (Fig. 8E). By 24 hpf, elongation continued and although leftward movement was observed, it was not as distinct as in the WT siblings (Fig. 8F). Together, these expression-based data suggest that embryonic loss of *pdgfra* function gives rise to abnormal endocardial migration during cardiac assembly.

## DISCUSSION

An RP2-mediated insertional mutagenesis system, which disrupts gene expression by truncating the native transcript, was used in this study to generate a zebrafish line with an insertional mutation in *pdgfra*. We demonstrated that this insertional mutation results in loss of endogenous tyrosine kinase domains, as a result of truncation of the native *pdgfra* mRNA. Moreover, the RP2 system simultaneously tags the truncated gene product with mRFP, allowing for spatio-temporal evaluation of protein expression. These tools facilitate characterization of *pdgfra* function in cardiac morphogenesis.

An in-cross of heterozygous GTB1300 gives rise to WT, heterozygous, mild and severe homozygous fish. As the heterozygous embryos are viable and grow to fertile adults, we believe that the mutant phenotype of this line would likely not be from a dominant negative effect. However, more experimental evidence is needed to support this conclusion. In a previously generated zebrafish *pdgfra^b^*^1059^ mutant line, a severe homozygous phenotype was not reported, which we observed in a small percentage (2.2%) of our GBT1300 homozygous mutants. Since we ruled out the presence of additional RP2 insertions and WT *pdgfra* transcripts, the low frequency of severe homozygotes may be explained by variation in the genetic background of each fish. A previous study demonstrated that the TU zebrafish genome is the most highly variable of the strains used for research. It was suggested that laboratory breeding of TU fish from a composite population may increase recombination events involving unique genotypic differences, resulting in hybrid offspring with even greater genetic – and possibly phenotypic – variability (Brown et al., 2012). We believe that these changes in the genetic background of the fish may partially account for the cause of observed differences between GBT1300 mild and severe homozygotes.

Phenotypes in the previously generated *pdgfra^b^*^1059^ have been proposed to be less severe than those in mouse *Pdgfrα* models, likely due to residual tyrosine kinase activity (Eberhart et al., 2008). In this study, we report a mutant zebrafish *pdgfra* line, GBT1300, where the molecular structure of the insertional mutation suggested that there would be no tyrosine kinase activity. However, similar to *pdgfra^b^*^1059^, most of our homozygous GBT1300 mutants presented a less severe phenotype than in mice. Nevertheless, there are several phenotypic parallels between murine models lacking *Pdgfrα* expression and zebrafish *pdgfra* mutants. For example, mice with loss of PDGFRα function also develop facial clefts. Cardiovascular defects in mice are more severe and they typically die after 10.5-11 days of embryonic development (Grüneberg and Truslove, 1960). In contrast, most homozygous GBT1300 larvae show comparatively mild cardiac phenotypes and can readily survive past embryogenesis and into larval stage (up to 14 dpf). Given the molecular nature of the GBT1300 mutation, we would offer an alternative explanation for the less severe phenotypes in zebrafish *pdgfra* mutants. Sequence and protein modeling analyses suggest that zebrafish *pdgfra* and *pdgfr beta* (*pdgfrb*) have a high degree of similarity, whereas their orthologues in mice and humans are less similar. Thus, we propose that Pdgfrb activity in zebrafish may compensate for some of the lost function in *pdgfra* mutants, resulting in the less severe phenotypes and longer survival of null individuals.

Although there is preliminary evidence of cardiac defects in mice and zebrafish (Price et al., 2001; Eberhart et al., 2008), the exact role of PDGFRα in cardiac development remains elusive. In this study, we demonstrate that loss of *pdgfra* function gives rise to abnormal cardiac morphology, primarily as a result of defective cardiac fusion. Nevertheless, specification, differentiation and initial migration of endocardial and myocardial precursors occur normally in GBT1300 mutants. Thus, until this developmental stage, endocardial and myocardial cells appear to be independent of Pdgfra function. Subsequent examination of heterozygous hearts at 30 hpf suggested Pdgfra-mRFP expression in the endocardium, rather than the myocardium, proposing a possible cell non-autonomous role of Pdgfra during myocardial fusion.

In zebrafish, endocardial cells migrate from the ALPM towards the midline slightly before myocardial cells do so (around 17 hpf). Once endocardial cells reach the midline, posterior then anterior myocardial fusion occurs around the centrally located endocardial precursors (Bussmann et al., 2007). Analysis of the GBT1300 mutants demonstrated defective endocardial migration to the midline, where cardiac assembly eventually occurs. In *cloche* mutants, which exhibit loss of endothelium (and thus, endocardium) (Stainier et al., 1995; Liao et al., 1997), myocardial fusion occurrence is relatively normal, with the exception of cardiomyocytes transiting from medial to angular movement throughout cardiac fusion (Holtzman et al., 2007). Thus, although endocardial signaling is required for angular movement of the myocardium, it is not necessary for the fusion of the bilateral cardiac fields. Accordingly, endocardial Pdgfra signaling in the GBT1300 mutants is likely not responsible for the abnormal fusion of the myocardium. Similarly, movement of endocardial precursor to the midline is not entirely dependent on medial migration of myocardial precursors (Palencia-Desai et al., 2015). Thus, the abnormal medial movement of endocardial precursors in the GBT1300 mutants is likely not a result of defective myocardial movement.

A study has shown that by 20 hpf, most endocardial cells are located ventral to the myocardium, especially in the lateral and posterior regions of the heart (Bussmann et al., 2007). Failure of the endocardium to move ventral to the myocardium can result in abnormal myocardial fusion. For example, in *tal1* mutants, endocardial cells remain located anterior to the myocardium at 20 hpf, leading to abnormal anterior fusion of the bilateral cardiac fields (Bussmann et al., 2007), a phenotype very similar to that observed in the GBT1300 mutants. Thus, it is possible that in GBT1300 mutants, abnormal migration of endocardial precursors to the midline results in failure of ventral movement, leading to physical restriction of the bilateral cardiac fields to fuse anteriorly. Our data highlights the need for greater insights into the molecular mechanisms of cardiac fusion and heart tube formation, which will contribute to our understanding of clinically pressing congenital cardiovascular abnormalities.

Complete abrogation of the pathways regulating nascent cardiac development almost always induces embryonic lethality in traditional vertebrate models and humans. However, zebrafish embryos exhibiting severe cardiomyopathies, as described here, can survive for days. This facilitates the analyses of genes that are otherwise embryonic lethal. Thus, characterization of the regulatory networks that orchestrate cardiac development in zebrafish is essential to elucidate the complete set of genes that regulate congenital heart malformations. Our results may have implications for the study of congenital heart malformations as failures of cardiac fusion can result in abnormal heart morphology, including cardia bifida and biconal heart. Moreover, the *pdgfra* mutants we describe here are also amenable to high-throughput screening to identify small molecules that can rescue aberrant cardiac fusion. This approach using zebrafish has clinical utility in stem cell-based cardiac regeneration and cell replacement/substation approaches.

## MATERIALS AND METHODS

### Ethics statement and zebrafish husbandry

All zebrafish (*Danio rerio*) experiments were conducted under St. Michael's Hospital Animal Care Committee (Toronto, Ontario, Canada) approved protocol ACC403. The zebrafish were housed in the Li Ka Shing Knowledge Institute (St. Michael's Hospital, Toronto, Ontario, Canada) research vivarium and maintained and staged as previously described (Westerfield, 1993). In short, the fish were housed under a 14 h light:10 h dark cycle at 28°C. Embryos were produced by pair mating and raised in 1× E3 embryo medium (5 mM NaCl, 0.17 mM KCl, 0.33 mM CaCl_2_, 0.33 mM MgSO_4_), in the presence of 0.003% 1-phenyl-2-thiourea to minimize pigmentation and permit more accurate examination. Strains used in this study included WT Tuebingen (TU) (Zebrafish International Resource Center, Eugene, OR, USA), *Tg(cmlc2:EGFP)* (Huang et al., 2003), *Tg(flk1:EGFP)* (Jin et al., 2005), and *Tg(sox10:EGFP)* (Wada et al., 2005). Transgenic lines were crossed with GBT1300 to generate *Tg(GBT1300;cmlc2:EGFP)* and *Tg(GBT1300;sox10:EGFP)*.

### Generation of gene-trap lines

Generation of gene-trap lines using the RP2 system was conducted as previously described (Clark et al., 2011). Briefly, newly fertilized WT TU embryos (at 1-cell stage) were co-injected with 1-2 nl of 50 ng/μl RP2 vector and 100 ng/μl Tol2 transposase mRNA. Chimeric F_0_ fish with green fluorescent protein (GFP) covering >80% of the body were raised to adulthood and outcrossed with WT TU fish. F_1_ progeny were screened for the presence of mRFP expression to identify germline transmission. GBT1300 was the line selected for this study. Embryos positive for mRFP were selectively raised and outcrossed with WT TU fish for several generations to obtain offspring with a single RP2 insertion.

### Identification and confirmation of the trapped gene

Two methods were used to identify the trapped gene in GBT1300: inverse polymerase chain reaction (PCR) and thermal asymmetric interlaced (TAIL) PCR. For inverse PCR, genomic DNA was obtained from ∼25 F_4_ GBT1300 heterozygous embryos and processed as previously described (Clark et al., 2011). In brief, approximately 3 μg of genomic DNA was digested using a cocktail of AvrII, XbaI and SpeI. 300 ng of the digested product was self-ligated in a 20 μl reaction with T4-DNA ligase (Roche) at 16°C overnight (O/N). 1 μl of the ligation solution was used as a template for inverse PCR to amplify the 5′ RP2 flanking sequence (*mRFP* side). Primary and nested PCR primers included 5R-mRFP-P1 and 5R-mRFP-P2 paired with INV-OPT-P1 and INV-OPT-P2 (Table S1), respectively (Clark et al., 2011). The PCR product was gel purified using a QIAquick Gel Extraction Kit (Qiagen), cloned using a TOPO TA Cloning kit (Invitrogen) and sequenced. The sequence was identified using Basic Local Alignment Search Tool (BLAST).

TAIL PCR uses one specific and one degenerate primer (DP) to amplify the trapped gene (Table S1). The TAIL PCR for detection of insertion sites was prepared from a previous procedure described by Parinov et al. (2004). In brief, genomic DNA was extracted from ∼25 F_5_ GBT1300 heterozygous embryos. Primary, secondary and tertiary nested PCR primers for the 3′ RP2 flanking region (*GFP* side) are 3R-GM2-P1, 3R-GM2-P2 and Tol2-ITR(L)-O1 (Table S1), respectively. These RP2-specific primers were used in combination with DP3 for each PCR reaction. Approximately 1 μg of genomic DNA was used for a 25 μl primary PCR reaction. Cycle settings were as follows. Primary: (1) 95°C, 2 min; (2) 95°C, 20 s; (3) 61°C, 30 s; (4) 70°C, 3 min; (5) repeat from ‘cycle 2’ 5 times; (6) 95°C, 20 s; (7) 25°C, 3 min; (8) ramping 0.3°C/s to 70°C; (9) 70°C, 3 min; (10) 95°C, 20 s; (11) 61°C, 30 s; (12) 70°C, 3 min; (13) 95°C, 20 s; (14) 61°C, 30 s; (15) 70°C, 3 min; (16) 95°C, 20 s; (17) 44°C, 1 min; (18) 70°C, 3 min; (19) repeat from ‘cycle 10’ 15 times; (20) 70°C, 5 min. 1 μl of a 1:20 dilution of the primary reaction was used as the template in the secondary reaction. Secondary: (1) 95°C, 2 min; (2) 95°C, 20 s; (3) 61°C, 30 s; (4) 70°C, 3 min; (5) 95°C, 20 s; (6) 61°C, 30 s; (7) 70°C, 3 min; (8) 95°C, 20 s; (9) 44°C, 1 min; (10) ramping 1.5°C/s to 70°C; (11) 70°C, 3 min; (12) repeat from ‘cycle 2’ 15 times; (13) 70°C, 5 min. (14) repeat from ‘cycle 2’ 15 times; (15) 70°C, 5 min. 1 μl of a 1:20 dilution of the secondary reaction was used as the template in the tertiary reaction. Tertiary: (1) 95°C, 2 min; (2) 95°C, 20 s; (3) 44°C, 1 min; (4) ramping 1.5°C/s to 70°C; (5) 70°C, 3 min; (6) repeat from ‘cycle 2’ 32 times; (7) 70°C, 5 min. The products from the tertiary PCR were gel purified using a QIAquick Gel Extraction Kit (Qiagen), cloned using a TOPO TA Cloning kit (Invitrogen) and sequenced. The sequence was identified using BLAST.

To confirm the locus of RP2 insertion, genotyping of F_5_ GBT1300 heterozygous fish was performed using a gene specific primer, g-F (intron 16), and an mRFP primer, 5R-mRFP-P2 (Table S1). The resulting amplicon was sequenced and analyzed. To check for additional RP2 insertions, F_5_ GBT1300 fish were in-crossed, and WT, heterozygous and homozygous embryos were sorted based on mRFP expression and phenotype at 96 hpf. Genomic DNA was extracted from each group (∼30 embryos/group) and the GFP fragment of the RP2 transposon was amplified using GFP-F and GFP-R primers (Table S1) (Ding et al., 2013). Moreover, fusion between the mRFP and the trapped gene must be in-frame to produce red fluorescence (Clark et al., 2011). To determine if the mRFP in the GBT1300 line is in-frame, cDNA from homozygous embryos was amplified using a gene-specific primer, RT-F (exon 15) and an mRFP primer, 5R-mRFP-P0 (Table S1). The resulting band was sequenced. All subsequent experiments were performed on F_5_ or higher generations.

### Reverse transcription and quantitative PCR

Total RNA from 96 hpf GBT1300 WT, heterozygous, mild and severe homozygous embryos (25/group) was purified using RNeasy mini kit (Qiagen) and cDNA was synthesized using random hexamer primers. Primers in exons flanking the RP2 insertion site (indicated in Fig. 1B) were used to test for the presence of endogenous product using reverse transcription (RT) and quantitative real-time (qRT) PCR. For RT-PCR, primers RT-F and RT-R (exon 23) were used (Table S1). As a control, *geranylgeranyltransferase type I beta subunit* (*pggt1b*) primers, *pggt1b* forward and *pggt1b* reverse (Eisa-Beygi et al., 2013), were used (Table S1). For qRT-PCR, *pdgfra* gene specific primers qRT-F (exon 16) and qRT-R (exon 17) were used (Table S1). The reactions were referenced to the *glyceraldehyde 3-phosphate dehydrogenase* (*gapdh*) transcript. The qPCR was performed with three technical replicates.

### Alcian Blue staining

To visualize cartilaginous craniofacial structures, larvae were stained with Alcian Blue, as previously described (Neuhauss et al., 1996). In brief, 96 hpf larvae were fixed with 4% phosphate-buffered paraformaldehyde (PFA) O/N, then washed several times with 1% phosphate-buffered saline with 0.1% Tween-20 (PBST). The fish were then bleached with 30% hydrogen peroxide for 2 h, rinsed with 1% PBST and stained with Alcian Blue solution (1% concentrated hydrochloric acid, 70% ethanol, 0.1% Alcian Blue) O/N. The larvae were cleared by rinsing with acidic ethanol for 4 h. Specimens were then rehydrated in acidic ethanol/water series and subsequently stored and imaged in 100% glycerol.

### Rescue

To synthesize *pdgfra* mRNA, *pdgfra* cDNA was amplified from a mixture of 24-96 hpf WT TU cDNA using pdgfra-F and pdgfra-R primers (Table S1), as previously described (Eberhart et al., 2008). The resulting amplicon was gel purified using a QIAquick Gel Extraction Kit (Qiagen), and a secondary PCR was conducted to amplify the gel purified product using pdgfra-F-T7 and pdgfra-R-T3 primers (Table S1), which contain T7 and T3 promoter sequences. The secondary amplified product was then used as a template for *in vitro* transcription using a mMessage mMachine T7 kit (Invitrogen) to generate *pdgfra* mRNA. The resulting *pdgfra* mRNA was injected into newly fertilized eggs collected from a heterozygous GBT1300 in-cross to rescue the homozygous phenotype.

### Whole-mount *in situ* hybridization

Whole-mount *in situ* hybridization (ISH) was performed as previously described (Thisse and Thisse, 2008), using *cardiac myosin light chain 2* (*cmlc2*), *ventricular myosin heavy chain* (*vmhc*) and *atrial myosin heavy chain* (*amhc*), *fetal liver kinase 1* (*flk1*), *mRFP* and *pdgfra* riboprobes. To make the *flk1* probe, *flk1* cDNA was amplified from a mixture of 24-96 hpf WT TU cDNA using flk1-F and flk1-R primers (Table S1). The resulting amplicon was gel purified using a QIAquick Gel Extraction Kit (Qiagen). A secondary PCR was conducted to amplify the gel-purified product using flk1-F-T7 and flk1-R-T3 primers (Table S1), which contain T7 and T3 promoter sequences. The secondary amplified product was then used as a template for *in vitro* transcription using 10× Dig RNA labeling mix (Roche) and T3 RNA polymerases (MEGAscript, Thermo Fisher Scientific) to generate the *flk1* riboprobe. The *mRFP* riboprobe was synthesized from pCR-mRFP plasmid using 10× Dig RNA labeling mix (Roche) and T7 RNA polymerases (MEGAscript), as previously described (Clark et al., 2011). To generate the *pdgfra* riboprobe, the same secondary amplified product used to generate *pdgfra* mRNA was used as a template for *in vitro* transcription using 10× Dig RNA labeling mix and T3 RNA polymerases (MEGAscript). Embryos subjected to ISH were cleared and imaged in 100% glycerol or benzylbenzoate:benzyl alcohol (Sigma).

### Microangiography

To check for the presence of blood circulation, 72 hpf larvae from a GBT1300 in-cross were anesthetized using 0.16 mg/ml tricaine and injected with FITC dextran (70 kD) through the common cardinal vein. The larvae were transferred to embryo water and allowed to recover O/N before being imaged.

### Whole-mount o-Dianisidine staining

Embryos were stained with o-Dianisidine (OD) (Sigma-Aldrich) to detect the presence of hemoglobinized blood. Briefly, larvae were fixed with 4% PFA O/N and subsequently stained with an OD solution [0.6 mg/ml o-dianiside, 0.01 M sodium acetate (pH 4.5), 0.65% hydrogen peroxide, and 40% ethanol] for 5 min in the dark. Embryos were washed with 1% PBST, then stored and imaged in 100% glycerol.

### Imaging

Bright-field and fluorescent images were captured using fluorescent dissection microscopy (Leica M205 FA) or confocal microscopy (Zeiss LSM 700) equipped with the appropriate filters. Embryos/larvae were anesthetized using 0.16 mg/ml tricaine methanesulfonate (Sigma-Aldrich), then embedded in 2.5% methyl cellulose (Sigma) or 1% low melting agarose (BioShop, Burlington, Canada) to obtain the desired orientation. ISH embryos were balanced between two glass capillary tubes to obtain dorsal views. The *Tg(GBT1300;cmlc2:EGFP)* beating heart videos were captured using Zeiss AxioObserver (Live Cell) (37 ms/frame). Genomic DNA was extracted from each embryo after imaging and PCR was performed using the following sets of genotyping primers: (1) gene-specific primers, pdgfra-g-F and pdgfra-g2-R (intron 16), and (2) *gfp*-specific primers, gfp2-F and gfp2-R (Table S1).
